# A dental perspective on the successes and limitations of the disaster victim identification response to the Nepal earthquake

**DOI:** 10.1080/20961790.2022.2034716

**Published:** 2022-03-10

**Authors:** Samarika Dahal, Gopal Kumar Chaudhary, Mani Raj Maharjan, Eugen Dolma Walung

**Affiliations:** a Department of Oral Pathology & Forensic Dentistry, Maharajgunj Medical Campus, Institute of Medicine, Kathmandu, Nepal; b Department of Forensic Medicine, Maharajgunj Medical Campus, Institute of Medicine, Kathmandu, Nepal

**Keywords:** Forensic sciences, earthquake, forensic dentistry, identification, Nepal

## Abstract

This article describes the forensic odontological analysis of the events of the 2015 Nepal earthquake. It identifies the problems encountered in the aftermath, lessons learned, and prospective future advances aimed at reducing the subjectivity in disaster victim identification (DVI). During a crisis, dental practitioners, particularly forensic odontologists, can make a substantial contribution to DVI, as highlighted in this article. It also promotes best practices in forensic dentistry that may be used by anyone in situations with few resources or people to deal with comparable scenarios.

## Key points

- Forensic odontologists recruited during the Nepal earthquake discuss their first-hand experience during the operation.
- The unique operation experience prepared them well to develop a protocol to effectively and efficiently identify an individual during such mass disasters.

## Introduction

Nepal is sandwiched between the Indian and Tibetan tectonic plates, making it an earthquake hotspot. Even though several high-profile disasters have dominated global headlines [1,2], the April 2015 Nepal earthquake, also known as the Gorkha earthquake, was extremely devastating, killing 9 000 people [2]. This massive earthquake struck Nepal’s mountainous terrain and measured 7.8 on the Richter scale. The earthquake revealed gaps in disaster preparedness in terms of management of the dead [3,4]. Many recommendations for disaster victim identification (DVI) have been created. The International Criminal Police Organization (INTERPOL) DVI guidelines, which are well accepted and adopted by most countries around the world [5], could be used as a benchmark against the manuscript to compare the response to issues such as lack of preparedness, improper recovery, and reconciliation challenges.

## Case details

Many victims of the earthquake were cremated in the Pashupatinath Temple’s ghats. Most bodies were identified by visual recognition and released by the police authorities from the site itself, due to an erroneous belief about disease spreading *via* dead bodies. Police officials cremated many bodies that were not scientifically identified.

The Department of Forensic Medicine (DoFM) at the Maharajgunj Medical Campus (MMC), Institute of Medicine, Kathmandu, Nepal, received 400 bodies [6] from the surrounding areas. At the time of the earthquake, the DoFM only had space to store 20 bodies at a time. Because of the large number of bodies received at the DoFM and the limited storage space available, they were aligned in the courtyard of the MMC’s basic science building. Later, with the help of the International Committee of the Red Cross (ICRC), additional cold storage arrangements were made to accommodate an increasing number of admissions up to 150.

Nepal police authorities gave the team permission to conduct examinations and gather information 3 days after the massive earthquake. The DVI operation began with the team members labelling and documenting the bodies, which were photographed by the police photographer with the support of volunteers from other groups. Meanwhile, Nepal police personnel documented and performed an inquest [6]. The authors, together with additional forensic professionals, conducted an external examination followed by a dental examination for all remains. On the 8th day of the DVI operation (2 May 2015), 23 bodies of victims of the Langtang avalanche were also brought to the MMC for identification.

### Body recovery phase

Search and rescue personnel who did not have forensic training oversaw the recovery of the dead due to the earthquake. The rescue officials entrusted to this assignment had little or no experience in search and rescue. Hence, the evidence was collected incorrectly, and body parts were recovered improperly.

None of the bodies had a documented recovery location. The body numbers were written on a piece of paper, which became indecipherable after contact with decomposition fluids or as a result of the cleaning process. None of the bodies were photographed at the recovery site. Many bodies were handed over to the families on the basis of visual recognition at the recovery site. Despite the large number of personnel participating, the recovery was poorly managed, producing many problems that obstructed identification efforts.

### The postmortem (PM) phase

Forensic experts conducted external examinations for all the deceased. Scientists from the Nepal Police Forensic Science Laboratory collected fingerprints and DNA samples from the unidentified bodies. Swabs for DNA profiling were collected from an incision made across the intercostal area, puncturing the lungs and allowing any uncontaminated blood to seep out. Forensic odontologists then performed a dental examination.

Because of a lack of qualified personnel and that Nepal only had two skilled forensic odontologists at that time, the dental examination was a significant and highly challenging task. ICRC PM forms in the Nepalese language were used to record all PM findings. Following initial training, members of the scene of crime officer (SOCO) team transcribed the PM analysis results. The MMC had no radiological equipment, so radiographs of the bodies could not be taken. Uninvited, many international experts and teams from all over the world arrived, dressed in clean uniforms with their national flags emblazoned on them. They were granted permission to collect DNA and dactylography samples. However, they were not involved in the identification process. ICRC, Nepal Red Cross Society, and the International Federation of Red Cross were among the international groups that offered aid and assistance.

The victims of the Langtang avalanche following the earthquake were eventually brought in for identification. Upper molar teeth were collected from these victims for DNA samples, which is now a standard procedure that was not done previously.

### The antemortem (AM) phase

Following the earthquake, no list of missing people was compiled, highlighting a flaw in disaster planning. In Nepal, the practice of storing dental records is still in its infancy. As a result, retrieving the AM data for Nepali victims following the earthquake was extremely challenging. The families were asked to provide dental records of missing relatives for AM data collection. Frequently, the families were unaware of recent dental work and so could not report it. The families who were aware of the dental treatment undertaken were often unable to identify the dentist, clinic location, or type of treatment. Even if the dentist was identified, the information gleaned from the records was insufficient. Had the dental data been properly stored and sent on time, there would have been an improved prospect of positive identification.

Obtaining AM data from numerous foreign embassies was also challenging, but deemed necessary to try to identify missing foreigners who were presumed dead. Messages did not always get through to the appropriate authorities, which caused delays in the AM data collection. Owing to miscommunication between the police, embassies, and the DoFM, some AM records were lost. Furthermore, the dental records retrieved were in a variety of languages, necessitating their translation into English. Even though many of the dental records were written in English, forensic personnel had difficulty interpreting the treatment undertaken because of different recording systems. There was some uncertainty with case files because the recording was incomplete and the transcribing AM team dentist failed to recognise the type of restorations present. Details such as the tooth surface that had been restored (e.g. mesial, occlusal) were not mentioned in some records. Only the type of tooth restoration was indicated. Sometimes, even the material used for crown restorations was not indicated. Another common charting error included misinterpretation of missing teeth (e.g. lower third molars interpreted as second molars). Details such as midline diastemata, spacing between the teeth, attrition, and descriptions of crowns and bridges were also commonly missing. The dental records that had been gathered were insufficient in number and details. Many dentists failed to provide radiographs, models, clinical pictures, and dental case files, resulting in inconsistencies in dental data recording. The AM data were gathered from only three embassies, whereas 22 countries reported missing people. Based on police reports of missing people from the respective embassies, 23 missing AM records were gathered. The initial stages of review of the dental records were complicated because they had not been filed alphabetically. Later, the DoFM and the ICRC collaborated to organise files alphabetically in a custom-made drawer. Additionally, a weekend fell in the middle of the workflow, lengthening the time it took to collect records from the embassies.

### Phase of reconciliation

In the first days after the earthquake, photographs of belongings were taken and pasted on a whiteboard, which was then placed in an open space to assist families to identify missing relatives. The police identified many bodies on their own and handed them over to the families despite the objections of the team members. As a result of this carelessness, much data useful for identification was overlooked.

A total of 365 bodies were positively identified using personal belongings, fingerprints, and in certain cases, DNA analysis. Because of a lack of reliable AM data, only six victims were identified based on dental evidence. The MMC’s cold storage facility still holds 13 unidentified bodies (10 males and three females) and 17 packets of body parts.

During the DVI operation, some forensic specialists appeared to be subject to cognitive bias. Bodies that were brought to the MMC were labelled with the victim’s putative name based on circumstantial evidence. It is possible that the putative identifications might have influenced subsequent analysis.

## Discussion

In a developing country such as Nepal, DNA testing may not be the best option for identifying victims because the possibility of a large number of victims entails a significant financial burden. Therefore, DNA-based identification is recommended only when dental and fingerprint methods cannot be used [7,8]. During this mass disaster, dental examination was one method used for identification. The use of odontological methods for identification has several advantages. It is simple to conduct, cost-effective, and a single distinguishing trait may facilitate identification [9].

The limitations of this method are that the comparison requires the mandated provision of AM documents. As was seen in this response, dental records for most Nepalese citizens did not exist or were untraceable. However, the foreign nationals’ AM records were traceable because of the dental record-keeping system in place in developed countries, enabling identification by dental/DNA methods.

Challenges faced:

- Dental evidence was not recorded, collected, or protected by trained personnel.
- Trained personnel and equipment were limited. With only two certified forensic officers on staff, effective, efficient, and quality-controlled dental identification was severely limited.
- The dental records retrieval process, which included collaborating with foreign embassies and storing AM dental data, was difficult. The collecting and storage of AM data became an enormous task.
- There was no adequate storage in the MMC for the deceased or for records. It was difficult to deal with the data because there was no way to digitise it or build an alphabetical master file for storage.
- There is a lack of a national tailored computer system for data entry. The response would have been much more effective if radiographic equipment and data management software had been available.
- The interpretation of AM records was problematic because they were written in several languages. Assistance was required for the translation of those records, which increased the amount of time it took for DVI operations to be completed.

Lessons learned:

- Systematic recording and collection of human remains with adequate storage is important, as rapid gathering and haphazard collection of the dead bodies led to the loss of loose teeth and the separation of heads from bodies.
- It is important to be aware of logistical challenges during the dental autopsy.
- The status and stock of emergency supplies should be monitored.

Considering the challenges that we faced in collecting and identifying the deceased resulting from this mass fatality event, we make the following recommendations to improve the process for future events:

- Prepare a directory of forensic odontology experts to establish better coordination and collaboration during the DVI procedures. It is proposed to prepare a dedicated dental DVI team in Nepal.
- Assess the strengths and limitations of recruited odontologists. During a major disaster, dental responders (forensic officers) and their teams may confront some problems as a result of their lack of expertise or experience in responding to disasters and mass fatality events, national practice differences, financial/resource considerations, the methods of recovery and management of the deceased and, on occasion, media pressure. Therefore, to cope with mass crisis circumstances, the recruited dentists should be evaluated ahead of time and trained to prepare them for this role.
- Develop ways to establish identity. In Nepal, the dental record storage system must be fully operational (it is expected to become mandatory in all Nepalese dental institutes and private clinics). This will allow a system to be set up to facilitate the swift retrieval of dental records in the event of a disaster.
- Adopt a uniform notation scheme and dental coding symbols. For the nationwide implementation of a standard notation system, an integrated instructional programme needs to be developed. This can be done in two stages: first, train the trainers, who in turn can train other potential team members.
- Incorporate software for dental identification. During a disaster, dental identification software allows for the electronic capture of data, which can then be catalogued and filtered to compare AM and PM information.
- Assemble a local search and recovery team that includes an anthropologist. The addition of an anthropologist to the local search and recovery team allows for a more detailed investigation. The anthropologist could be useful in identifying differences between bone, teeth and other objects and also for associating bones with sets of remains. In countries where human resources are not abundant, one anthropologist may not be able to do both recovery and analysis. In these situations, the focus should be on analysis.
- Create an efficient and quality-regulated procedure to reduce unnecessary workflow strain. Any anxiety during the identifying procedure invites mistakes.
- Grant an individual AM team leader responsibility for all AM dental records to improve communication during the collection of AM data.
- Encourage the use of cost-effective methods during the identification process. The use of DNA testing imposes a financial burden on the country. The variety of DNA tests available and the specialised interpretation involved requires specific expertise. Therefore, DNA testing should only be used when dental comparisons and fingerprinting are inconclusive.

## Conclusion

The purpose of this article was to share the specific challenges faced in Nepal following the 2015 earthquake. These included limitations of resources and planning, and poorly organised recovery of the remains of the victims. We have highlighted lessons learned and shared recommendations so that others might avoid similar problems. We stress the importance of establishing organised, standardised methods that facilitate victim identification.
